# Protein Synthesis Dependence of Growth Cone Collapse Induced by Different Nogo-A-Domains

**DOI:** 10.1371/journal.pone.0086820

**Published:** 2014-01-29

**Authors:** Richard Manns, Andre Schmandke, Antonio Schmandke, Prem Jareonsettasin, Geoffrey Cook, Martin E. Schwab, Christine Holt, Roger Keynes

**Affiliations:** 1 Department of Physiology, Development and Neuroscience, University of Cambridge, Cambridge, United Kingdom; 2 Brain Research Institute, University of Zurich and Department of Health Sciences and Technology, Swiss Federal Institute of Technology, Zurich, Switzerland; National Institutes of Health (NIH), United States of America

## Abstract

**Background:**

The protein Nogo-A regulates axon growth in the developing and mature nervous system, and this is carried out by two distinct domains in the protein, Nogo-A-Δ20 and Nogo-66. The differences in the signalling pathways engaged in axon growth cones by these domains are not well characterized, and have been investigated in this study.

**Methodology/Principal Findings:**

We analyzed growth cone collapse induced by the Nogo-A domains Nogo-A-Δ20 and Nogo-66 using explanted chick dorsal root ganglion neurons growing on laminin/poly-lysine substratum. Collapse induced by purified Nogo-A-Δ20 peptide is dependent on protein synthesis whereas that induced by Nogo-66 peptide is not. Nogo-A-Δ20-induced collapse is accompanied by a protein synthesis-dependent rise in RhoA expression in the growth cone, but is unaffected by proteasomal catalytic site inhibition. Conversely Nogo-66-induced collapse is inhibited ∼50% by proteasomal catalytic site inhibition.

**Conclusion/Significance:**

Growth cone collapse induced by the Nogo-A domains Nogo-A-Δ20 and Nogo-66 is mediated by signalling pathways with distinguishable characteristics concerning their dependence on protein synthesis and proteasomal function.

## Introduction

The protein Nogo-A has been identified as an important regulator of development, plasticity and regeneration in the vertebrate nervous system [1]. Nogo-A (1200 aa, 200 kD) is a member of the Reticulon family of proteins (Reticulon-4, Rtn4), so-called due to the presence of a C-terminal 200 aa RTN homology domain comprising two >35 aa hydrophobic stretches, and the *Nogo/Rtn4* gene gives rise to 3 main isoforms (A, B, C), of which Nogo-A is the largest [2]. Consistent with its proposed role as a negative regulator of axon growth, Nogo-A is expressed at the cell surface [3] and causes collapse of a wide variety of growth cones *in vitro*. Further studies have identified key domains of the protein that elicit collapse [4], [5], and two domains in particular have been implicated, Nogo-66 and Nogo-A-Δ20. Nogo-66 is a 66 amino acid domain that, together with flanking hydrophobic regions, is a component of the RTN homology domain in the C-terminus of all Nogo isoforms [6]. Nogo-66 collapse-inducing activity is associated with high-affinity binding to its receptors NgR1 [7], [8], which forms a complex with the transmembrane proteins LINGO1, and p75 or TROY [1], [9]–[11]. Nogo-66 can also bind to the paired immunoglobulin-like receptor PirB [11]. Receptor binding activates the Rho/Rho-associated coiled-coil containing protein kinase (ROCK) pathway, resulting in growth cone collapse through RhoA signalling and destabilization of the actin cytoskeleton [1], [12], [13].

The other growth cone collapse-inducing domain, Nogo-A-Δ20 (NiGΔ20) comprises residues 544–725 of (rat) Nogo-A, and is a component of the extracellular N-terminal domain (residues 1–979). The cognate receptor(s) for Nogo-A-Δ20 and the detailed signalling pathways that lead to collapse are less well characterized. Both integrins [14] and a G protein-coupled receptor [15] have been implicated. Like Nogo-66, Nogo-A-Δ20 activates the RhoA-ROCK pathway [1], [4], [12]. Moreover Nogo-A-Δ20 signalling has been shown to inactivate Rac, a GTPase whose regulatory functions on the cytoskeleton oppose those of Rho [12], [16]. Nogo-A-Δ20-induced growth cone collapse also requires endocytosis of a Nogo-A-Δ20/receptor complex that is retrogradely transported to the cell body in signalling endosomes containing activated Rho. This process is clathrin-independent and mediated by the pinocytotic chaperone protein Pincher [5]. Since Nogo-A-Δ20 endocytosis is directly linked to reduced levels of phosphorylated neuronal cyclic AMP response element-binding protein (CREB), the process may be a mechanism for Nogo-A-Δ20 to modulate expression of genes that regulate neuronal growth [5].

Protein synthesis in the growth cone provides a further important influence on the signalling events that mediate axon guidance and regeneration [17], [18]. For example growth cone collapse caused by the axon guidance protein sema3A [19] has been shown to be protein synthesis-dependent [17], [20], and this dependence varies according to the concentration of sema3A to which growth cones are exposed [21]. The protein synthesis dependence of growth cone collapse induced by Nogo-A-Δ20 and Nogo-66 is unknown, and this study was therefore undertaken to elucidate this aspect of Nogo-mediated growth cone signal transduction. Our main finding is that Nogo-A-Δ20-induced collapse is dependent on protein synthesis whereas Nogo-66-induced collapse is independent of protein synthesis. This indicates that these two Nogo-A domains engage differing signalling pathways that mediate growth cone collapse.

## Results

**Figure 1:**
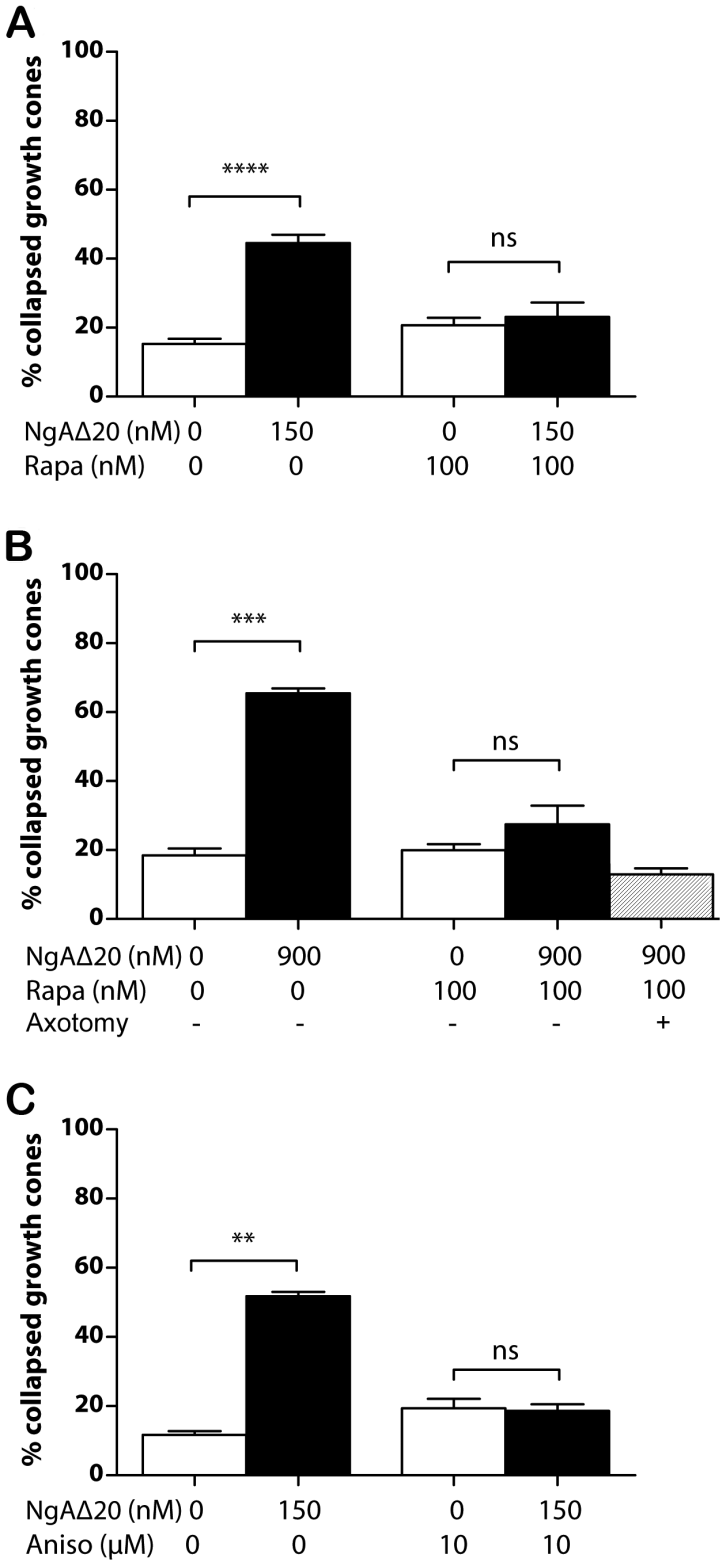
Dependence of Nogo-A-Δ20-induced growth cone collapse on protein synthesis. **A/** Nogo-A-Δ20–induced collapse remains at control levels in the presence of 150 nM rapamycin. **B/**Nogo-A-Δ20-induced collapse remains at control levels in the presence of 900 nM rapamycin. Collapse is not affected by axotomy prior to rapamycin exposure. **C/**Nogo-A-Δ20–induced collapse remains at control levels in the presence of 10 µM anisomycin.

The dependence of Nogo-A-Δ20-induced growth cone collapse on protein synthesis was examined using explanted chick dorsal root ganglion (DRG) neurons growing in the presence of NGF (40 ng/ml). As shown in Figure 1A, Nogo-A-Δ20 (150 nM) caused ∼45% of all growth cones to collapse 30 minutes after addition to the cultures, compared with ∼15% collapse in control cultures (addition of PBS). In the presence of 100 nM rapamycin to block protein translation through mTOR complex 1, Nogo-A-Δ20-induced collapse was reduced to control levels (addition of PBS and rapamycin but not Nogo-A-Δ20). When the Nogo-A-Δ20 concentration was increased 6-fold to 900 nM, collapse increased to ∼65%, and this was again prevented by rapamycin (100 nM), which reduced collapse to control levels (Figure 1B). Inhibition of protein translation by the ribosomal inhibitor anisomycin (10 µM) also reduced Nogo-A-Δ20-induced collapse to control levels (Figure 1C).

**Figure 2:**
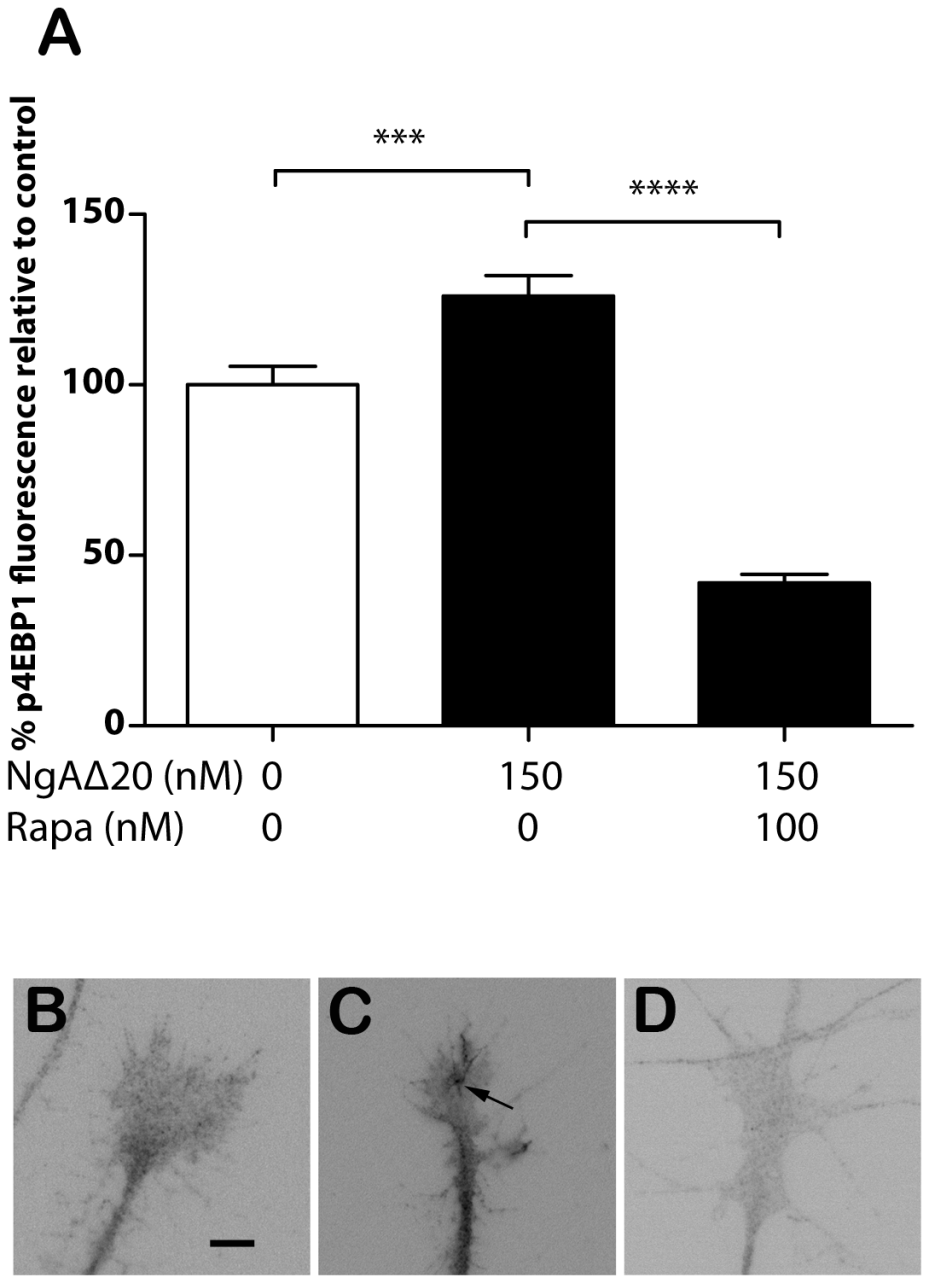
mTOR activity after application of Nogo-A-Δ20. **A/** Phosphorylation of eIF4E-BP1 is increased 15 minutes after application of 150 nM Nogo-A-Δ20, and is inhibited by rapamycin. **B/−D/**Examples of growth cones (fluorescence intensity normalized and contrast inverted) exposed respectively to control (**B/**), 150 nM Nogo-A-Δ20 (**C/**, arrow indicates region of growth cone with increased signal), and both 150 nM Nogo-A-Δ20 and 100 nM rapamycin (**D/**).

As a measure of protein synthesis in these experiments we confirmed that application of 150 nM Nogo-A-Δ20 increases growth cone phosphorylation of eukaryotic initiation factor 4E binding protein 1 (eIF4E-BP1), a key downstream target of mTOR complex 1. Within 15 minutes of Nogo-A-Δ20 application phosphorylation increased significantly over control (Figure 2A,B). As expected, the combination of 150 nM Nogo-A-Δ20 and 100 nM rapamycin reduced the phosphorylation signal significantly compared with both control and Nogo-A-Δ20 alone, indicating a basal level of mTOR activity in these cultures. To confirm that rapamycin acts on growth cones independently of the neuronal nucleus, the assay was repeated using axons acutely severed from their cell bodies; rapamycin still prevented growth cone collapse of axotomized axons at 30 minutes (Figure 1B). A further control experiment, using a separate batch of Nogo-A-Δ20, showed that the proportion of growth cones of axotomized axons that collapse in response to Nogo-A-Δ20 (900 nM) is 44.1% +/−2.1 s.e.m.; this was the same as for intact axons using this batch of Nogo-A-Δ20 (46.3% +/−4.6 s.e.m.). Additionally, we used azidohomoalanine (AHA) and Click chemistry to show that axonal protein synthesis increases in growth cones after exposure to Nogo-A-Δ20 in response to mTOR activity. Acutely severed DRG axons were incubated for 1 hour in methionine-free medium with 100 µM AHA, a methionine analogue that can be covalently coupled to an alkyne-conjugated fluorochrome via Click chemistry [22], [23], before incubation for 1 hour with 150 nM Nogo-A-Δ20 or both Nogo-A-Δ20 and 100 nM rapamycin. Analysis of AHA-labelled proteins by SDS gel electrophoresis confirmed that Nogo-A-Δ20 induces a rapamycin-inhibitable increase in labeled proteins within 1 hour (Figure S1).

**Figure 3:**
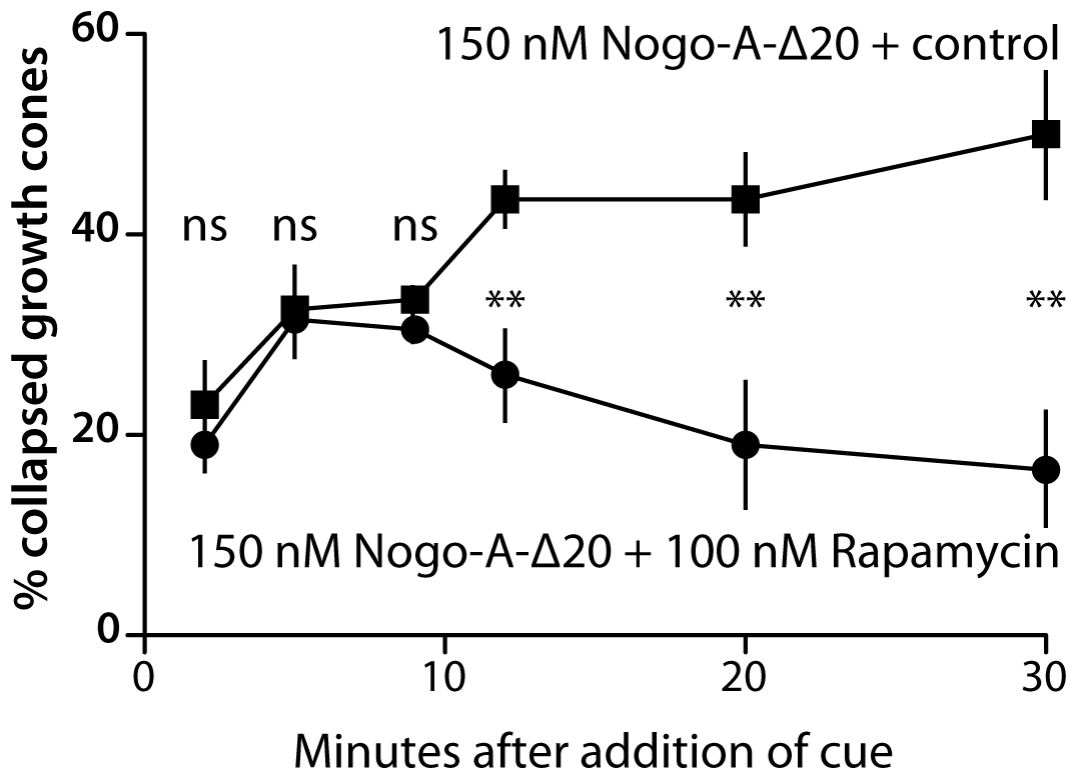
Time course of Nogo-A-Δ20-induced collapse and its dependence on protein synthesis. The degree of collapse over time following addition of 150-A-Δ20 in the presence (filled circles) and absence (filled squares) of 100 nM rapamycin. Between 2 and 5 minutes post-exposure, collapse increased significantly to ∼30% with and without rapamycin. From 12 minutes post-exposure, rapamycin-exposed growth cones progressively recovered from collapse, while growth cones treated with rapamycin vehicle control maintained the extent of collapse at >40%.

To assess the time course of Nogo-A-Δ20-induced growth cone collapse, DRG axons were exposed to 150 nM Nogo-A-Δ20 for periods between 2–30 minutes before fixation, with and without addition of rapamycin (100 nM). At time points 5 and 9 minutes post-exposure to Nogo-A-Δ20, collapse increased to ∼30% both in the presence and absence of rapamycin (Figure 3). Beyond 9 minutes, collapse further increased towards ∼50% in the absence of rapamycin, while it fell to below ∼20% in the presence of rapamycin (see Discussion).

**Figure 4:**
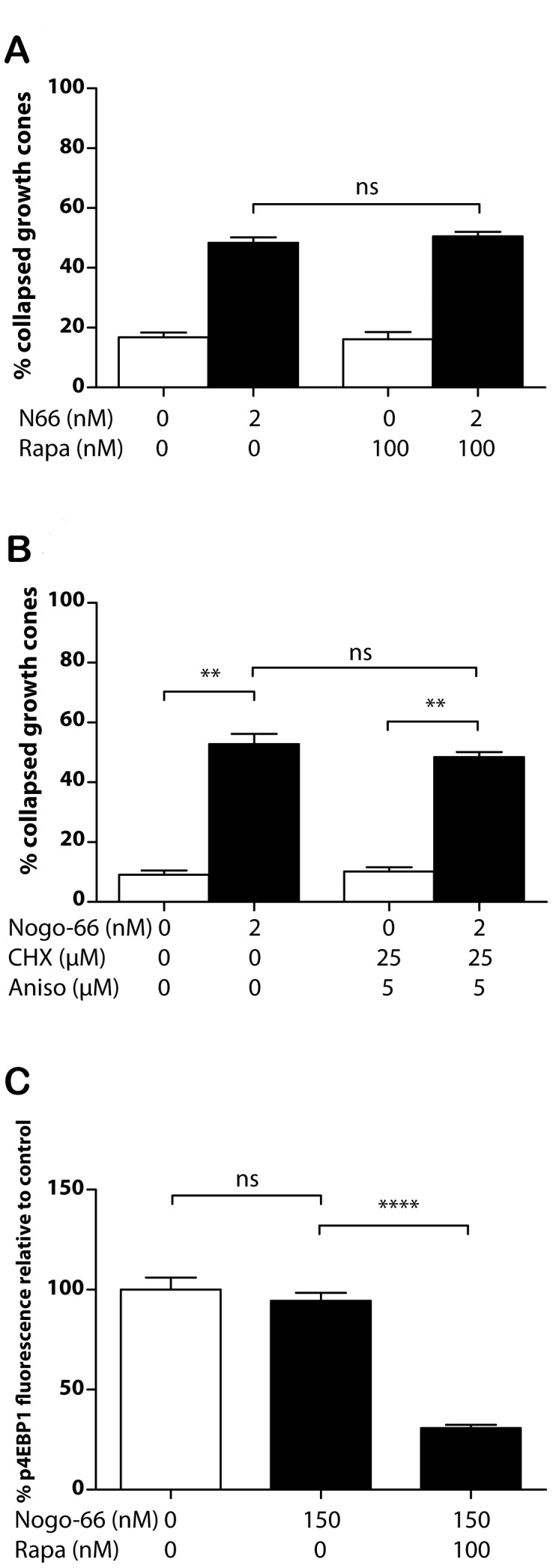
Dependence of Nogo-66-induced growth cone collapse on protein synthesis. **A/** Nogo-66-induced collapse remains in the presence of 150 nM rapamycin. **B/**Nogo-66-induced collapse also remains in the presence of 2 µM cycloheximide and 5 µM anisomycin. **C/**Phosphorylation of eIF4E-BP1 is not affected by application of 2 nM Nogo-66, but is inhibited by 100 nM rapamycin.

The dependence of Nogo-66-induced growth cone collapse on protein synthesis was then examined in the same experimental system. As shown in Figure 4A, the presence of 2 nM Nogo-66 was sufficient to elicit ∼50% growth cone collapse after 30 minutes, while neither rapamycin nor the combination of anisomycin and cycloheximide inhibited collapse (Figure 4B). This indicates that, in contrast to Nogo-A-Δ20, Nogo-66 induces collapse independently of protein synthesis. Consistent with this conclusion, 15 minutes after exposure of axons to 2 nM Nogo-66 there was no significant change in the level of phosphorylated eIF4E-BP1 in growth cones, whereas 100 nM rapamycin in addition to Nogo-66 reduced phosphorylation signal levels as expected (Figure 4C).

**Figure 5:**
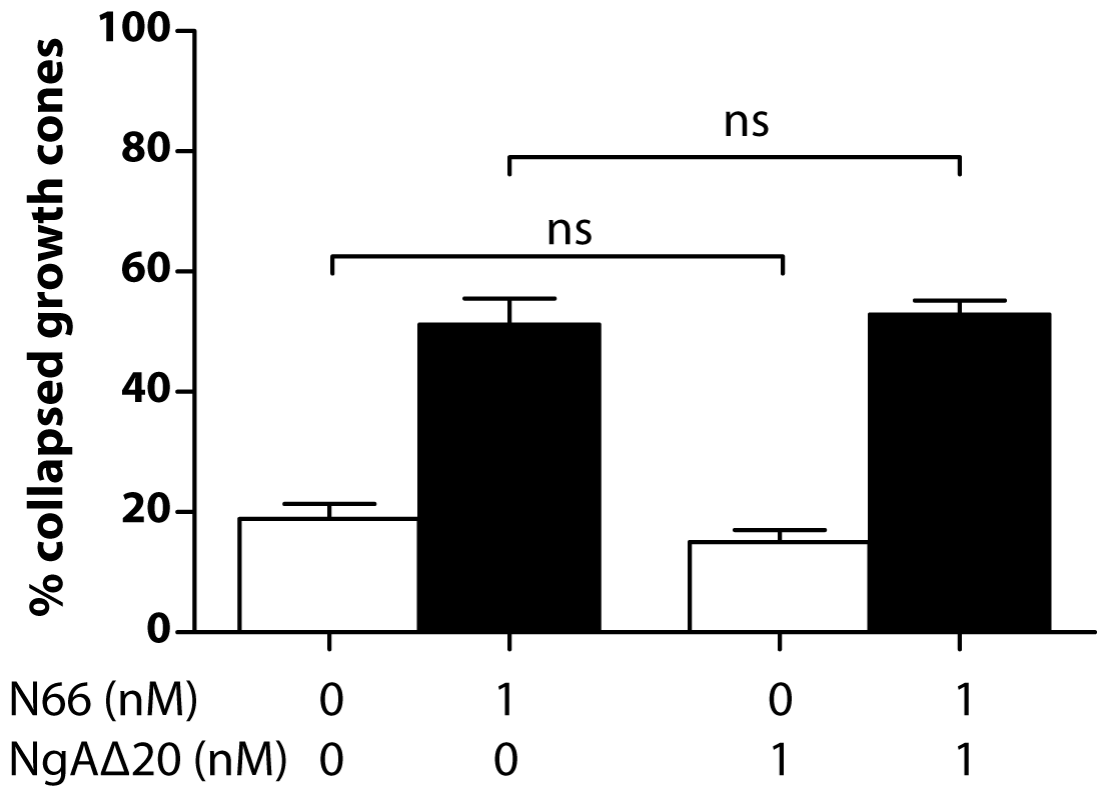
Growth cone collapse in the presence of equal concentrations of Nogo-66 and Nogo-A-Δ20. 1-66 induces significant growth cone collapse, and this is not altered by the presence of 1 nM Nogo-A-Δ20.

It is not clear whether Nogo-A-Δ20 and Nogo-66 signal co-operatively or independently *in vivo*, and we therefore tested whether synergy between Nogo-A-Δ20 and Nogo-66 is detectable when both collapse-inducing molecules are applied together at the same concentration. Nogo-66 is known to have a higher specific activity for growth cone collapse than Nogo-A-Δ20 [4]. We therefore chose a concentration of Nogo-66 (1 nM) that induces ∼50% collapse and tested this in combination with Nogo-A-Δ20 at the same concentration, allowing possible synergy to be detectable. As expected, addition of 1 nM Nogo-A-Δ20 did not significantly increase collapse over control levels. Moreover, combining the two molecules, both at 1 nM, did not increase collapse beyond ∼50% (Figure 5), indicating no synergy at this concentration.

**Figure 6:**
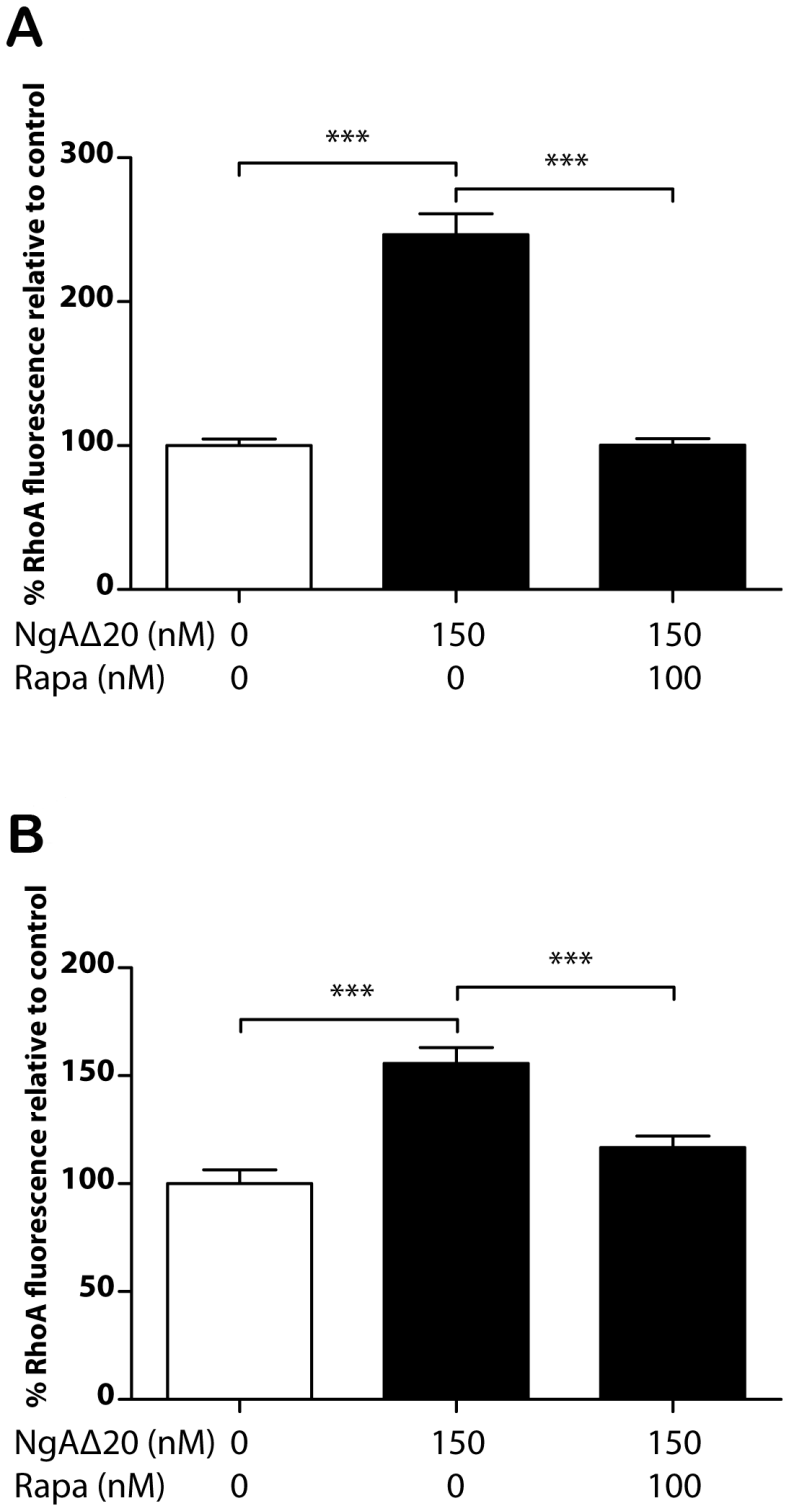
RhoA Levels after application of Nogo-A-Δ20. **A/, B/** Levels of RhoA in growth cones detected by anti-RhoA monoclonal antibodies SC-179 (**A/**) and 26C4 (**B/**) after 15 minute exposure to control (PBS), 150 nM Nogo-A-Δ20 and both 150 nM Nogo-A-Δ20 and 100 nM rapamycin, respectively. RhoA increases significantly within 15 minutes of exposure to Nogo-A-Δ20, but rapamycin prevents this increase.

Further experiments were carried out to investigate related signalling pathways in the growth cone that might be engaged by Nogo-A-Δ20. Growth cone collapse in response to the repulsive cue Sema3A has been shown to be mediated by local synthesis of RhoA [20], and we tested whether the Nogo-A-Δ20-induced increase in RhoA activity [4], [12] is regulated similarly (Figure 6). RhoA levels were measured 15 minutes after exposure to 150 nM Nogo-A-Δ20 by growth cone immunofluorescence using two different monoclonal anti-Rho antibodies. In both cases fluorescence increased significantly in response to Nogo-A-Δ20 and this was prevented by prior addition of 100 nM rapamycin (Figure 6), indicating a requirement for local protein synthesis of RhoA for Nogo-A-Δ20-responsivity. We also tested the role of cGMP signalling in Nogo-A-Δ20-induced collapse, using 1H-[1], [2], [4]oxadiazolo[4,3-a]quinaloxin-1-one (ODQ, 500 nM) to inhibit soluble guanylyl cyclase and cGMP signalling, and found that this did not inhibit collapse (Figure S2).

**Figure 7:**
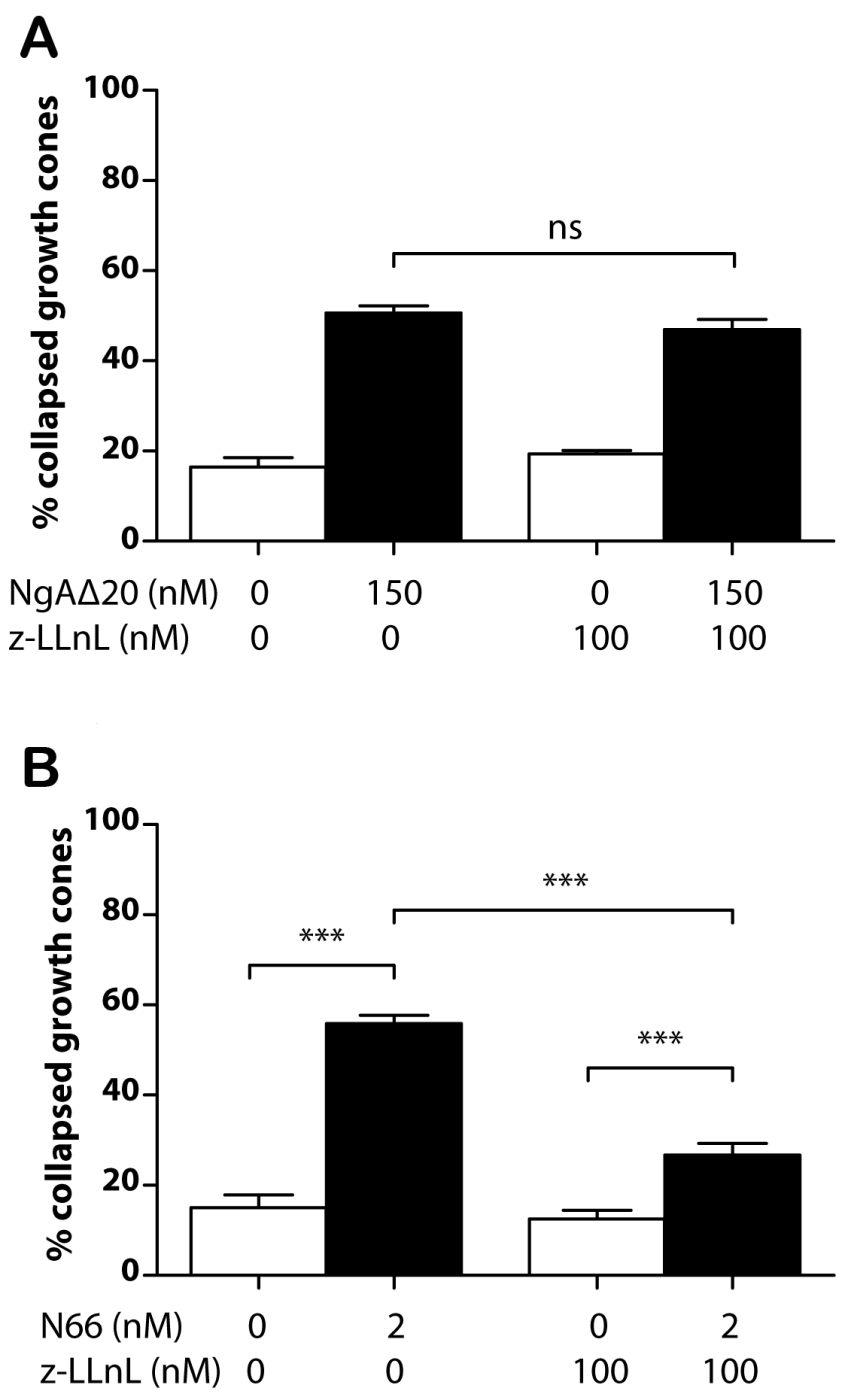
Proteasome inhibition and Nogo-induced growth cone collapse. **A/** Proteasome inhibition with Z-LLnL (LLnL) does not inhibit the collapse-inducing activity of 150 nM Nogo-A-Δ20. **B/**Proteasome inhibition significantly inhibits collapse-inducing activity of 2 nM Nogo-66 (N66).

Last, we assessed the involvement of proteasomal function and ubiquitin-tagged protein degradation in Nogo-A-induced growth cone collapse, testing Nogo-A-Δ20 and Nogo-66 in separate experiments. Proteasomal catalytic site inhibition with N-acetyl-L-leucyl-L-leucyl-L-norleucinal (LLnL, 100 nM) had no significant effect on Nogo-A-Δ20-collapse-inducing activity (Figure 7A). However the same concentration of proteasomal inhibitor reduced Nogo-66-induced collapse by ∼50% (Figure 7B).

## Discussion

Our experiments using chick DRG axons indicate several differences in the growth cone signalling pathways engaged by the Nogo-A collapse-inducing domains Nogo-A-Δ20 and Nogo-66. Nogo-A-Δ20-induced collapse is dependent on local protein synthesis/translation, as for other guidance cues such as sema3A, slit2 and netrin 1 [17], [18], [21]. However, in contrast to sema3A-induced collapse [21] there is no evidence that collapse induced by high concentrations of Nogo-A-Δ20 is independent of protein synthesis; at both lower (150 nM) and higher (900 nM) concentrations, Nogo-A-Δ20-induced collapse is reduced to control levels by blockade of mRNA translation. Two further distinctions between Nogo-A-Δ20- and sema3A-induced signalling in the growth cone are also notable. First, Nogo-A-Δ20-induced collapse involves Pincher-mediated endocytosis whereas sema3A-induced collapse does not [5], and second, inhibition of soluble guanylyl cyclase inhibits collapse induced by sema3A [24]–[26] but not by Nogo-A-Δ20 (this study).

The time course of Nogo-A-Δ20-induced collapse shows that some growth cones collapse rapidly following initial exposure to Nogo-A-Δ20 (within 10 minutes), and this takes place whether or not rapamycin is also present (Figure 3). This may reflect the existence of a sufficient pool of pre-existing protein in these growth cones to elicit collapse without the requirement for *de novo* synthesis, and such rapid collapse is plausible as a physiological mechanism during axon guidance *in vivo*. Alternatively, it may reflect a delay in the onset of action of rapamycin compared with the initiation of Nogo-A-Δ20-induced collapse. Our findings additionally indicate that the subsequent rapamycin-sensitive phase of Nogo-A-Δ20-induced growth cone collapse (10–30 minutes) is independent of the cell body, since it also occurs in acutely axotomized neurites. This is consistent with the study of Joset et al. [5] showing the requirement for Pincher-mediated endocytosis in mediating Nogo-A-Δ20-induced collapse. Using compartmentalized (rat DRG) cultures, distal neurites but not proximal neurites or neuronal cell bodies were found to accumulate Nogo-A-Δ20-containing endosomes within 30 minutes of Nogo-A-Δ20-exposure, while the latter sites contain them only at later time points [5].

In sharp contrast to Nogo-A-Δ20, we find that Nogo-66-induced growth cone collapse takes place independently of protein synthesis, as confirmed by the absence of phosphorylation of eIF4E-BP1 after Nogo-66 exposure. Like Nogo-66, collapse due to high concentrations of sema3A (>500 ng/ml) is independent of protein synthesis, and the latter pathway has been shown to involve GSK-3β activation [21]. In this respect it is interesting that a recent study [27] has shown that myelin-associated inhibitors of axon growth induce phosphorylation and inactivation of GSK-3β, rather than activation. Alabed et al. used a DRG axon outgrowth assay rather than a growth cone collapse assay, and more detailed investigation of growth cone regulation by GSK-3β in response to Nogo-A-derived peptides is therefore warranted.

The finding that *de novo* synthesis of RhoA in the growth cone is required for Nogo-A-Δ20-induced collapse provides another contrast with Nogo-66-induced collapse, which also involves RhoA activation [1], [12], [13] but does not require protein synthesis (Figure 4). A further difference between the two collapse-inducing pathways is that proteasomal inhibition reduces Nogo-66- but not Nogo-A-Δ20-induced collapse. A possible mediator here is the scaffold protein Plenty of SH3 (POSH [28]), which is downstream of Nogo-66/PirB signalling. This has E3 ubiquitin ligase activity, although the target ubiquitinated downstream of Nogo-66 is unknown.

While our results indicate that Nogo-66 induces growth cone collapse independently of mTOR, Nogo-66 has been shown to activate mTOR in the context of stem cell differentiation, regulating both astrocyte differentiation from neural progenitor cells [29] and ES cell pluripotency via regulation of the transcription factor nanog [30]. Moreover the synthesis of both glutamate receptors [31] and GABA_B_ receptors [32] is suppressed by NgR1 signalling via the mTOR pathway, again presumably through Nogo-66 rather than Nogo-A-Δ20.

Regarding the role of Nogo-A in axon growth regulation *in vivo*, Schwab and colleagues have speculated that the primary function of Nogo-66/NgR signalling may concern axon guidance, since this system possesses higher specific activity for growth cone collapse than Nogo-A-Δ20 [4]. While Nogo-A-Δ20 may have a similar role, our evidence indicates that the two domains do not synergize with respect to growth cone collapse when used together at concentration (1 nM) that induces ∼50% collapse with Nogo-66 alone (Figure 5). The operating concentration range of Nogo-A *in vivo* remains unknown, however, and our results do not exclude the possibility that domain synergy takes place at concentrations higher than 1 nM. There is also evidence that Nogo-A-Δ20 exerts an additional sustained influence on neuronal gene expression mediating long-term suppression of axon growth [1], [4], [5], [33]. This is supported by the study of Chivatakarn et al. [34], who showed that myelin-induced chronic inhibition of axon outgrowth *in vitro* is independent of NgR1 signalling. Our findings revealing several differences in the growth cone signalling pathways engaged by these two Nogo-A domains are consistent with this proposed functional separation.

## Materials and Methods

Nogo-66-FC (as a disulfide-linked homodimer) was purchased from R&D Systems and Nogo-A-Δ20 was purified as described previously [4]. Briefly, BL21/DE3 E. coli were transformed with the pET28 expression vector (Novagen) containing the sequence of the recombinant His−/T7-tagged protein and cultured at 37°C until an OD of 0.8 AU. 1 M IPTG was added for 2 h at 30°C to induce protein expression. After cell lysis with BugBuster Protein Extraction Reagent (Novagen) the fusion protein was purified using Co^2+^-Talon Metal Affinity Resin (Takara Bio Inc.).

F-12 medium, penicillin/streptomycin and DMEM medium were obtained from PAA, and B27 supplement, L-15 and Click-iT® AHA Alexa Fluor® 488 protein synthesis reagents from Invitrogen. Insulin/transferrin/selenite (ITS+3), NGF, glutamine, laminin from mouse sarcoma, poly-L-lysine, anisomycin, rapamycin and cycloheximide were purchased from Sigma-Aldrich, and Borosilicate cover-slips from VWR International. 1H-[1], [2], [4]oxadiazolo[4,3-a]quinaloxin-1-one (ODQ) was obtained from Cayman Chemical, and N-acetyl-L-leucyl-L-leucyl-L-norleucinal (LLnL) from Sigma. Anti-p-4EBP1 antibody was purchased from Cell Signaling Technology, and Alexa Fluor 594 secondary antibody from Life Technologies. Anti-RhoA monoclonal antibodies SC-179 and 26C4 were obtained from Santa Cruz Biotechnology.

Coverslips for chick DRG explants were cleaned in acid and ethanol, and flamed immediately before use. DRG explants were dissected from E7 chick embryos; no ethical approval was required for this procedure under English law since it took place within the first two-thirds of the chick embryo incubation period [The Guidance on the Operation of the Animals (Scientific Procedures) Act 1986 (amended 2013)]. Coverslips were coated in 100 µg/ml poly-L-lysine for 1 h and then 20 µg/ml laminin for 1 h, both steps at 38°C. E7 DRGs were dissected in medium and grown overnight at 38°C in DMEM and NGF (80 ng/ml) in 5% CO_2_. Inhibitors and inhibitor controls were introduced 1 min prior to Nogo-A peptide or PBS/vehicle controls, and cultures were incubated at 38°C in 5% CO_2_ for 30 min. Axonal transection was carried out adjacent to the body of the DRG using a hypodermic needle. Explants were fixed with a solution of 4% w/v formaldehyde and 15% w/v sucrose in PBS for 2 h at room temperature. The levels of collapse in blind-coded samples were assessed by phase contrast microscopy; growth cones with two or fewer filopodia were designated as collapsed, and at least 6 fields of view were assessed for each DRG culture. Data groups were compared using the non-parametric Mann-Whitney *U*-test and the Kruskal-Wallis ANOVA test; all percentage values are means. For each data point growth cone numbers averaged 150, minimum 50, from at least 3 cultures. Quantitative immunofluorescence was performed on cultures grown in 160 ng/ml NGF (a high concentration to maintain a spread growth cone morphology in all samples so that comparative measurements could be made [20], [35]). Anti-p-4EBP1 antibody was used at 1∶100, and its fluorescence signal in growth cones was assessed 15 minutes after application of Nogo-A-Δ20. Each growth cone was imaged under white light and then under fluorescence illumination. The white-light images were used to define the growth cone outline, excluding the axon and central zone of the growth cone but including the lamellipodia and filopodia (peripheral zone) up to the growth cone transition zone. The central zone was excluded due to the variable thickness of this part of the growth cone, causing a significant source of error in a two-dimensional analysis. The fluorescence intensity was measured as an average across the growth cone area thus defined, as described by Campbell and Holt [17]. Inhibition of protein synthesis in growth cones was monitored using the Click-iT® AHA Alexa Fluor® 488 protein synthesis assay following manufacturer’s instructions.

## Supporting Information

Figure S1 **AHA-TAMRA labeling of protein synthesis after exposure of DRG neurons to Nogo-A-Δ20. A/**TAMRA-labeled newly synthesized protein during 1 h exposure to control (C), 150 nM Nogo-A-Δ20 (N) and both Nogo-A-Δ20 and 100 nM rapamycin (NR). The rate of protein synthesis increases markedly across a range of molecular weights after exposure to Nogo-A-Δ20, and this increase is prevented by rapamycin indicating its dependence on mTOR. **B/**Colorized version of image A, showing the gradient spectrum in the lower right-hand corner (black/blue low intensity, white/red high intensity); there is a marked increase in protein synthesis due to Nogo-A-Δ20 (N) compared with control (C), which is inhibited by rapamycin (NR). **C/**Quantification of the total fluorescence in each lane.
(TIF)

Figure S2 **Soluble guanylyl cyclase and Nogo-A-Δ20-induced growth cone collapse.** Inhibition of soluble guanylyl cyclase with 1H-[1], [2], [4]oxadiazolo[4,3-a]quinaloxin-1-one (ODQ, 500 nM) does not affect Nogo-A-Δ20-induced growth cone collapse.
(TIF)
