# Supplementary figures and images for: Protein Synthesis Dependence of Growth Cone Collapse Induced by Different Nogo-A-Domains
Source: PLoS One. 2014 Jan 29;9(1):e86820. doi: 10.1371/journal.pone.0086820 (PMC3906062; doi:10.1371/journal.pone.0086820)

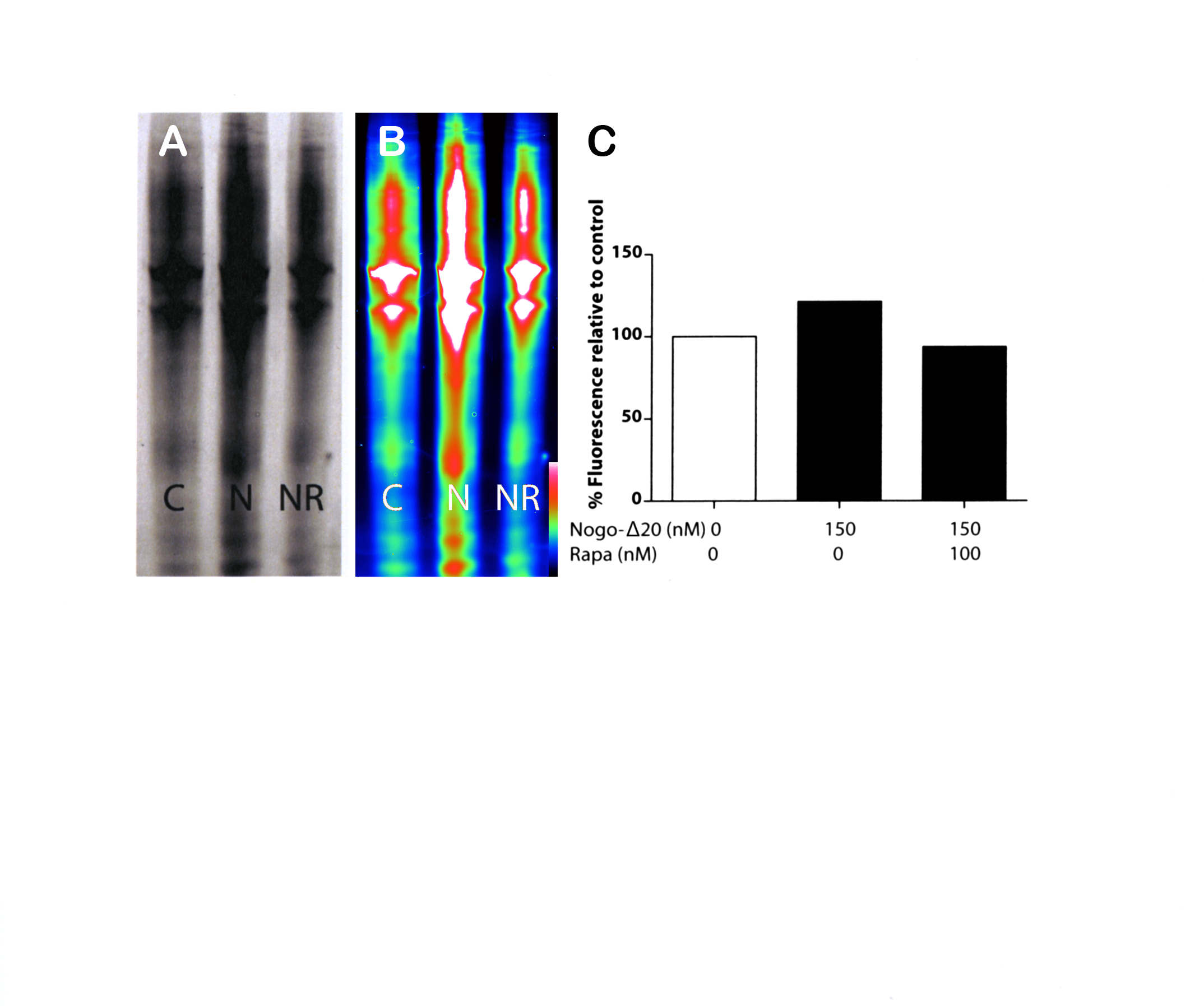

Supplement: Figure S1 — AHA-TAMRA labeling of protein synthesis after exposure of DRG neurons to Nogo-A-Δ20. A/TAMRA-labeled newly synthesized protein during 1 h exposure to control (C), 150 nM Nogo-A-Δ20 (N) and both Nogo-A-Δ20 and 100 nM rapamycin (NR). The rate of protein synthesis increases markedly across a range of molecular weights after exposure to Nogo-A-Δ20, and this increase is prevented by rapamycin indicating its dependence on mTOR. B/Colorized version of image A, showing the gradient spectrum in the lower right-hand corner (black/blue low intensity, white/red high intensity); there is a marked increase in protein synthesis due to Nogo-A-Δ20 (N) compared with control (C), which is inhibited by rapamycin (NR). C/Quantification of the total fluorescence in each lane. (TIF) [file pone.0086820.s001.tif]

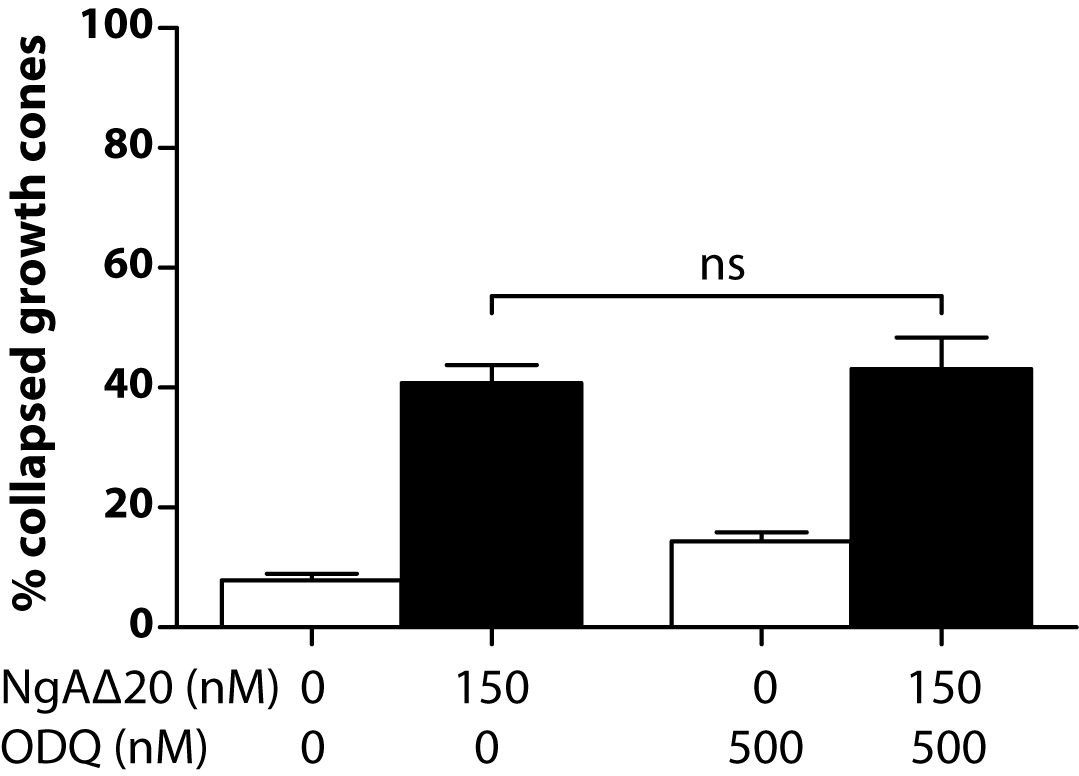

Supplement: Figure S2 — Soluble guanylyl cyclase and Nogo-A-Δ20-induced growth cone collapse. Inhibition of soluble guanylyl cyclase with 1H-[1], [2], [4]oxadiazolo[4,3-a]quinaloxin-1-one (ODQ, 500 nM) does not affect Nogo-A-Δ20-induced growth cone collapse. (TIF) [file pone.0086820.s002.tif]
